# High-frequency oscillations in scalp EEG mirror seizure frequency in pediatric focal epilepsy

**DOI:** 10.1038/s41598-019-52700-w

**Published:** 2019-11-12

**Authors:** Ece Boran, Johannes Sarnthein, Niklaus Krayenbühl, Georgia Ramantani, Tommaso Fedele

**Affiliations:** 1 Klinik für Neurochirurgie, UniversitätsSpital & Universität Zürich, Zürich, Switzerland; 2 Zentrum für Neurowissenschaften Zürich, ETH Zürich, Zürich, Switzerland; 3 Pädiatrische Neurochirurgie, Universitäts-Kinderspital Zürich, Zürich, Switzerland; 4 Neuropädiatrie, Universitäts-Kinderspital Zürich, Zürich, Switzerland; 5 Institute of Cognitive Neuroscience, Higher School of Economics - National Research University, Moscow, Russian Federation

**Keywords:** Epilepsy, Neurophysiology, Diagnostic markers, Electroencephalography - EEG, Epilepsy

## Abstract

High-frequency oscillations (HFO) are promising EEG biomarkers of epileptogenicity. While the evidence supporting their significance derives mainly from invasive recordings, recent studies have extended these observations to HFO recorded in the widely accessible scalp EEG. Here, we investigated whether scalp HFO in drug-resistant focal epilepsy correspond to epilepsy severity and how they are affected by surgical therapy. In eleven children with drug-resistant focal epilepsy that underwent epilepsy surgery, we prospectively recorded pre- and postsurgical scalp EEG with a custom-made low-noise amplifier (LNA). In four of these children, we also recorded intraoperative electrocorticography (ECoG). To detect clinically relevant HFO, we applied a previously validated automated detector. Scalp HFO rates showed a significant positive correlation with seizure frequency (R^2^ = 0.80, *p* < 0.001). Overall, scalp HFO rates were higher in patients with active epilepsy (19 recordings, *p* = 0.0066, PPV = 86%, NPV = 80%, accuracy = 84% CI [62% 94%]) and decreased following successful epilepsy surgery. The location of the highest HFO rates in scalp EEG matched the location of the highest HFO rates in ECoG. This study is the first step towards using non-invasively recorded scalp HFO to monitor disease severity in patients affected by epilepsy.

## Introduction

In invasive EEG, high frequency oscillations (HFO) have been proposed as a reliable biomarker of epileptogenic tissue, bearing the potential to guide the surgical treatment of drug-resistant focal epilepsy^1–4^. In addition, invasive HFO were shown to mirror disease activity in this patient subgroup, as they increase after antiepileptic drug (AED) tapering^5,6^. However, invasive recordings carry a considerable sampling bias, providing a high resolution for brain regions sampled by subdural or depth electrodes, but potentially missing epileptic activity that derives from other brain regions. Most importantly, invasive EEG recordings entail risks of morbidity that are only justified for selected epilepsy surgery candidates. These considerations have instigated a shift of HFO research towards non-invasive recordings, particularly towards the widely accessible scalp EEG, aiming to develop potential surrogates of invasive explorations and, ultimately, valid tools of drug and disease monitoring relevant to a large number of patients affected by epilepsy.

In scalp EEG, HFO have been recently associated with the seizure onset zone in drug-resistant focal epilepsy^7–9^. Furthermore, scalp HFO have been linked to the severity of disease in several pediatric epilepsy syndromes, such as infantile spasms^10^, atypical benign partial epilepsy^11^, and Rolandic epilepsy^12^, with scalp HFO rates mirroring seizure frequency in the latter study^12,13^. Moreover, a reduction of scalp HFO rates in response to treatment has been reported for children with continuous spike-and-wave during sleep^14^, West syndrome^10^, and atypical benign partial epilepsy^11^. Whether scalp HFO rates correlate with seizure frequency in drug-resistant focal epilepsy and whether a treatment response in terms of scalp HFO reduction can be observed with epilepsy surgery awaits confirmation. Most importantly, the relation of scalp HFO to invasive HFO is still under debate^15^. Although some studies have linked scalp HFO to invasive HFO in consecutive or simultaneous EEG recordings^7–9,16^ no study has longitudinally analyzed scalp as well as invasive HFO in the context of drug-resistant focal epilepsy so far. Thus, the clinical relevance of scalp HFO for therapy monitoring, particularly for the assessment of epilepsy surgery outcomes, remains to be evaluated.

Scalp HFO are hard to detect, and their analysis in a clinical setting is particularly challenging in terms of time and effort. The feasibility of detection for scalp HFO, corresponding to invasive HFO, has been attested under favorable signal-to-noise ratio (SNR) conditions^17,18^. In this line, we have previously demonstrated that a low-noise amplifier (LNA) significantly enhances the SNR in the HFO spectral range, thus improving HFO detection in the intraoperative electrocorticography (ECoG) compared to a commercial device (CD)^19,20^. Since HFO detection by human visual review is subjective as well as time-consuming, we previously developed an automated detector and validated it for the ECoG against seizure outcome^3,20^. The validity of this approach for scalp HFO detection in a clinical setting remains, however, to be determined.

In this study, we prospectively recorded pre- and postsurgical scalp EEG in children with drug-resistant focal epilepsy undergoing epilepsy surgery and used the LNA to improve HFO detectability as well as an automated detector to ensure a prospective, bias-free definition of clinically relevant HFO. We hypothesized that scalp HFO rates would match seizure frequency, correspond to invasive HFO, and decrease after successful epilepsy surgery, thus constituting not only a valuable biomarker for surgical planning but also a valid indicator of disease severity.

## Methods

### Patients

We prospectively enrolled 27 consecutive children and adolescents with drug-resistant focal epilepsy that underwent presurgical evaluation, surgical therapy and postsurgical follow-up at the cooperating institutions of the University Children’s Hospital Zurich, University Hospital Zurich, and Swiss Epilepsy Center, between August 2016 and September 2019. We selected 12 recordings from 11 patients (7 male) for further analysis that fulfilled the following inclusion criteria: scalp EEG (1) recorded at high sampling frequency (>2000 Hz), (2) containing at least 10 min of NREM sleep, and (3) recorded at >2 h from the most recent seizure, as well as resective epilepsy surgery. In all cases, we could form a clear hypothesis regarding the localization of the epileptogenic zone based on electro-clinical findings and the presence of an MR-visible lesion before the scalp EEG recording with the LNA.

Seizure frequency, as a measure of epilepsy severity, was assessed by long-term video-EEG or seizure diaries at the time of the pre- or postsurgical scalp EEG. Postsurgical seizure outcome was portrayed according to the ILAE scale. Epilepsy substrates were determined by histopathology.

### EEG recordings

#### Scalp EEG

All patients underwent routine pre- and postsurgical scalp EEG, including NREM sleep epochs, with electrodes placed according to the 10–20 system. The scalp EEG was recorded by a standard CD (Deltamed, Neurofile^®^), with an input noise level of ~21 nV/√Hz. In addition to standard clinical investigations and in parallel to the CD, we connected an 8-channel custom-made LNA with an input noise level of ~2.3 nV/√Hz. The LNA is battery powered, which prevents interference with the CD. Given the limited number of available LNA channels, we connected four adjacent electrodes over the presumed epileptogenic zone and four homologous electrodes over the contralateral hemisphere. Impedances were typically around 1 kΩ. Data were acquired at 10 kHz and down-sampled to 2 kHz for further processing.

#### ECoG

Intraoperative ECoG was recorded in four patients using either standard 1 × 6 subdural electrode strips or high-density 4 × 8 subdural electrode grids (AdTech Medical^®^). The strip electrodes had a contact exposure diameter of 5.0 mm and an inter-electrode distance of 10 mm. The grid electrodes had a contact exposure diameter of 2.3 mm and an inter-electrode distance of 5 mm. ECoG was recorded from different locations before and after surgical resection, and the electrode position was carefully documented by the surgeon and the pediatric neurologist in charge of the case. The placement of electrodes was guided solely by the clinical question. We used a needle electrode connected to the dura as the reference. We recorded ECoG with a Nicolet amplifier (Nicolet^®^ CSeries: Natus, Pleasanton, PA, USA; 16-bit ADC, sampling rate 2 kHz, 1–800 Hz passband). The signal was subsequently transformed to a bipolar montage.

FR detected with electrode strips of 10 mm contact spacing have proven sufficient for outcome prediction in several past studies^2,3,20–23^. High-density electrode grids with 5 mm contact spacing have been recently shown to improve FR detection and outcome prediction^23^. The ECoG coverage and spatial sampling were thus sufficient for intraoperative FR detection in our study.

#### Data selection

Scalp EEG was recorded while patients took afternoon naps. We selected exclusively NREM sleep intervals, since HFO rates are higher during NREM compared to REM sleep^24^. In both scalp EEG and ECoG, intervals with visible artifacts and channels with continuous interference were excluded from further analysis. The resulting data (mean duration of 27 ± 13 min per patient) was divided into 5 min epochs.

#### Automated HFO detection

HFO detection was performed with a previously validated automated detector^3,4,20^. The detection algorithm uses the instantaneous power spectrum as computed by the Stockwell transform in a three-stage workflow (Supplementary Material, Supplementary Fig. S1). During HFO detection, an amplitude threshold (Th_amp_) is computed to characterize the background activity. The detection was performed for HFO both in the ripple (80–250 Hz) and in the fast ripple (FR) band (250–500 Hz). This procedure ensured a prospective, bias-free definition of a clinically relevant HFO in both scalp EEG and ECoG.

#### Visual artifact rejection

**Figure 1:**
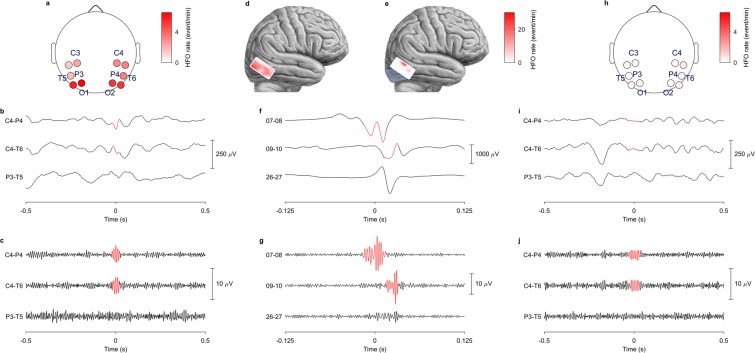
HFO rates for Patient 1. HFO rates and their localizations in presurgical scalp EEG (**a**–**c**), intraoperative ECoG (**e**–**g**) and postsurgical scalp EEG (**h**–**j**) in Patient 1 with a right occipital epileptogenic zone and resection. HFO rates and filtered signals are reported in the ripple band (80–250 Hz) for the scalp EEG (**b**,**c**,**i**,**j**) and in the fast ripple band (FR, 250–500 Hz) for the intraoperative ECoG (**f**,**g**). (**a**) Presurgical scalp EEG recorded with the low-noise amplifier (LNA). Circles indicate selected bipolar channels and color code the HFO rate in events/min, according to the scale on the right of the panel. HFO rates are higher over the affected (right) hemisphere. (**b**) Full-band EEG, as recorded from the bipolar channel C4-P4 showing HFO (in red), the adjacent bipolar channel C4-T6, also showing HFO, and the contralateral bipolar channel P3-T5, showing no HFO. (**c**) EEG filtered in the HFO band, recorded from the same bipolar channels as in (**b**), showing HFO in the bipolar channel C4-P4 and the adjacent bipolar channel C4-T6, but not on the contralateral bipolar channel P3-T5. (**d**) Pre-resection ECoG. A 4 × 8 subdural grid electrode was placed on the resection margin. The HFO rate detected in bipolar channels is color-coded in events/min, according to the scale on the right of the panel. (**e**) Post-resection ECoG. The resection area is shaded in light blue. Residual HFO are shown on the resection margin. The HFO rate detected in bipolar channels is color-coded in events/min, according to the scale on the right of the panel. The patient had two recurrent seizures in the 18 months since surgery under unchanged antiepileptic drug dosage (ILAE 3). (**f**) Full-band ECoG, as recorded from the bipolar grid channels showing HFO, from the adjacent bipolar channels, also showing HFO, and from remote bipolar channels, showing no HFO. (**g**) ECoG filtered in the HFO band, recorded from the same bipolar channels as in (**f**). Please note the different time scale applied for scalp EEG and ECoG, as depicted below the respective traces. (**h**) Postsurgical scalp EEG recorded with the LNA. Circles indicate selected bipolar channels and color code the HFO rate in events/min, according to the scale on the right of the panel. HFO rates are higher over the affected (right) hemisphere, as in the presurgical scalp EEG (**a**), but have considerably decreased after surgery. (**i**) Full-band EEG, as recorded from the bipolar channel C4-P4 showing HFO (in red) as well as from the adjacent bipolar channel C4-T6 and the contralateral bipolar channel P3-T5, showing no HFO. (**j**) EEG filtered in the HFO band, recorded from the same bipolar channels as in (**i**), showing HFO in the bipolar channel C4-P4, but not in the adjacent bipolar channel C4-T6 or the contralateral bipolar channel P3-T5.

Following the automated HFO detection in both scalp EEG and ECoG, we performed visual artifact rejection (Supplementary Fig. S1). Batches of EEG recordings from different patients were presented to the reviewer in random order. Thus, the reviewer was blinded both to the timing of the EEG recording and to the seizure frequency at the time of the recording. For each putative HFO, an observer inspected the signal in a 0.3 s window around the event in the full band and filtered in the HFO band. Putative HFO that the observer perceived as artifacts were excluded from further analysis. For scalp EEG recordings, artifacts were rejected by visual inspection from a single observer. For ECoG recordings, a second expert observer reviewed ambiguous events. Of the putative HFO detected automatically, 18% were excluded by visual artifact rejection. It should be noted that this step in the workflow did not detect HFO, but only rejected artifacts. HFO examples and rates are depicted in Fig. 1.

### Clinical validation of HFO

**Figure 2:**
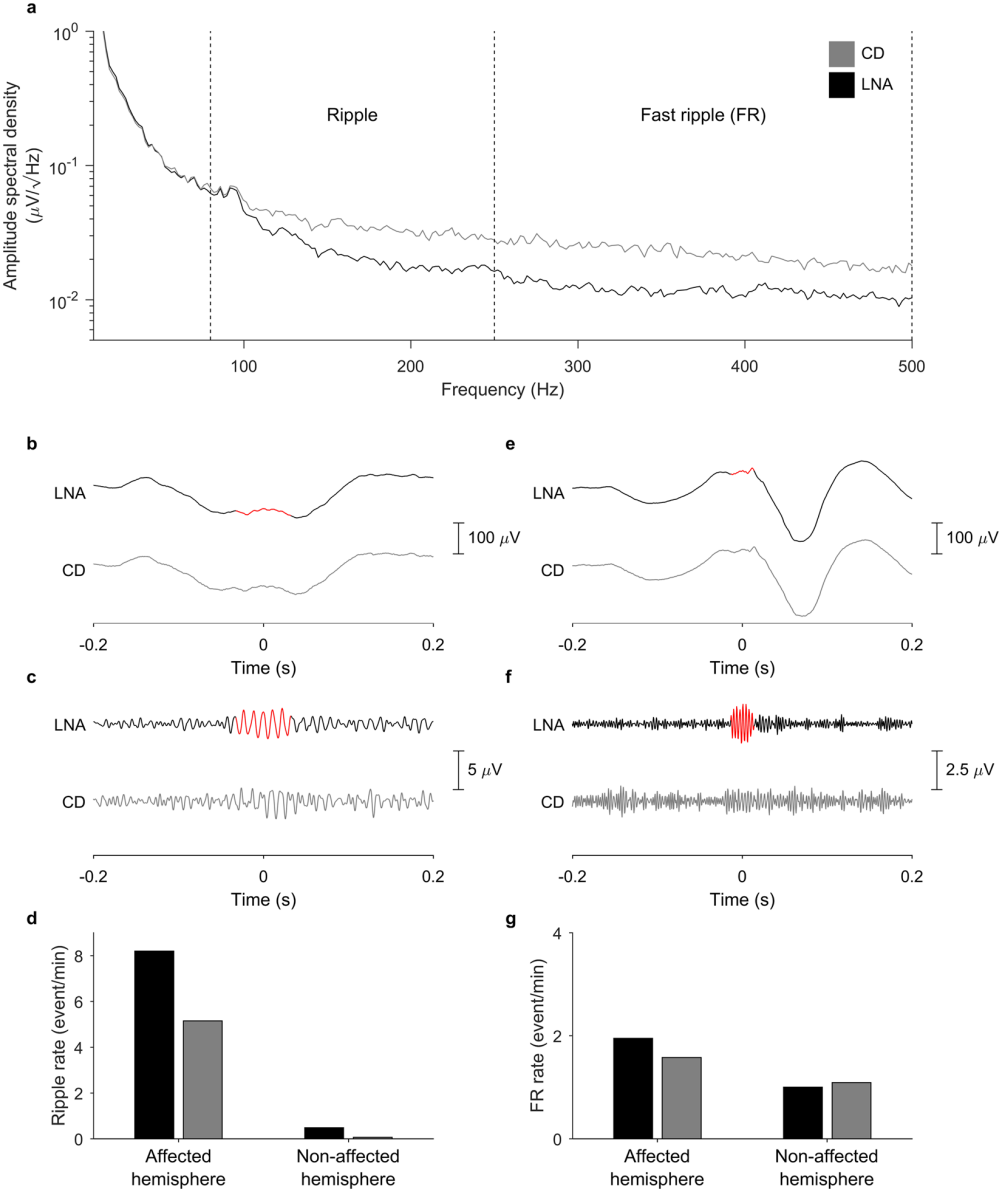
Noise level in scalp HFO recording for Patient 2. (**a**) In the linear amplitude spectral density above 100 Hz, the noise floor of the low-noise amplifier (LNA, black) was lower than that of the commercial device (CD, gray). The LNA is thus more sensitive to events of lower amplitude. This feature allows the LNA to detect a higher number of HFO in the ripple band (depicted in red in panels (b,c) as well as in the fast ripple (FR) band (depicted in red in panels (**e**,**f**) that went undetected in the CD recording (gray). (**d**,**g**) Overall ripple (**d**) and FR (**g**) rates were higher for LNA (black) than for CD (gray) over the affected hemisphere (*p* < 0.001 for HFO ripples, Wilcoxon matched-pairs signed rank test).

Automated HFO detection, visual validation, and analysis were performed blinded to clinical data. HFO were not used for clinical decision-making. We analyzed HFO in different frequency bands, depending on the EEG recording scale. For ECoG, the clinical validation of HFO was performed in the FR band, in line with previous studies^3,22^. For scalp EEG, the clinical validation of HFO was performed in the ripple band, since FR did not provide clinically relevant information in the exemplary case of Patient 2, as demonstrated in Fig. 2. If the scalp EEG HFO rate exceeded the rate threshold, we considered the patient to show HFO.

We defined as true positive (TP) a scalp EEG showing HFO in patients with active epilepsy (all patients before surgery; ILAE > 1 after surgery). We defined as false positive (FP) a scalp EEG showing HFO in seizure-free patients after surgery (ILAE 1). We defined as false negative (FN) a scalp EEG showing no HFO in patients with active epilepsy. We defined as true negative (TN) a scalp EEG showing no HFO in seizure-free patients. The positive predictive value (PPV) was calculated as PPV = TP/(TP + FP), the negative predictive value (NPV) as NPV = TN/(TN + FN), and the accuracy = (TP + TN)/N, whereas N signified the total number of recordings.

### Statistics

The correlation between HFO rate and seizure frequency was estimated using linear regression with ordinary least squares. We compared HFO rates between recordings with the Wilcoxon rank sum and matched-pair signed rank tests. To test case-wise changes in HFO rate and seizure frequency between pre-and postsurgical recordings, we used the chi-squared test. For recording level statistics, we computed the optimal rate threshold using receiver operating characteristic (ROC) curve analysis. Statistical significance was established at *p* < 0.05. We used the Wilson score to estimate 95% confidence intervals (CI).

### Ethics

The collection of patient data and the scientific analysis were approved and performed conform to the guidelines and regulations of the ethics committee (Kantonale Ethikkommission Zürich, KEK-ZH PB-2016-02055), and all patients and their parents gave written informed consent.

## Results

### Scalp HFO detection

Scalp HFO rates were higher for the affected than for the non-affected hemisphere (*p* = 0.0003, Wilcoxon matched-pairs signed rank test) in the ripple band, but not in the FR band. We therefore considered only scalp HFO in the ripple band as relevant for further analysis. This point is illustrated in Fig. 2 that presents the detected scalp HFO and their distribution between hemispheres in the ripple and FR bands in Patient 2.

It should be noted that, in presurgical scalp EEG, the two patients with deep lesions (Patient 3 & 4) located in the medial temporal region, showed HFO over the affected hemisphere at much lower rates (0.26–0.48 HFO/min) than in patients with superficial frontal, temporal or occipital lesions (0.62–11.18 HFO/min). Across all scalp EEG recordings, the amplitude threshold for HFO detection (Th_amp_) did not significantly differ between hemispheres, thus confirming overall good signal quality.

### Scalp HFO rates mirror epilepsy severity

The clinical features of our patients and the findings of scalp EEG and ECoG performed before, during, and after surgery are given in Table 1. Figure 1 illustrates the scalp HFO rate, as recorded by the LNA before and after surgery, in the exemplary case of Patient 1. The rate threshold computed with the ROC curve was 0.25 HFO/min.

**Table 1:** Patient characteristics.

Patient	Age, sex	Etiology	Seizure frequency pre	Resection	Follow-up period	Seizure frequency post	ILAE outcome	Scalp EEG pre			ECoG pre	ECoG post	Scalp EEG post		
								affected hemisphere		non-affected hemisphere			affected hemisphere	non-affected hemisphere	
			(seizures/month)		(months)	(seizures/month)		sites	mean rate (HFO/min)		maximal rate (HFO/min)		sites	mean rate (HFO/min)	
1	4, f	Sturge Weber syndrome	30	R lateral posterior temporal & lateral occipital	20	0.2	3	C4,O2,P4,T6	4.01	3.48	31.73	4.62	C4,O2,P4,T6	0.43	0.18
2	5, m	FCD 1a	180	L medial/lateral anterior temporal	27	180	5	C3,F7,T3,T5	7.03	0.45			C3,F7,T3,T5	3.57	0.85
2	7, m	FCD 1a	180	L temporo-posterior, occipital, parietal	4	0	1	C3,F7,T3,T5	3.57	0.85			C3,F7,T3,T5	0.52	0.18
3	10, m	diffuse astrocytoma	0.5	R medial anterior temporal	14	1	3	F4,F8,T4,T6	0.26	0.39			F4,F8,T4,T6	0.23	0.21
4	3, m	mMCD	2	R medial/lateral anterior temporal	14	150	5	F4,F8,T4,T6	0.48	0.17			C4,F4,F8,T4	2.36	2.21
5	13, m	cavernoma	8	L dorsal medial/lateral prefrontal	5	0	1	C3,F3,Fp1	1.68	0.34			C3,F3,F7,Fp1	0.10	0.04
6	15, m	DNET	12	R medial/lateral anterior temporal	5	0	1	C4,F8,T6	0.63	0.16			C4,F8,T4,T6	0.25	0.21
7	14, f	ganglioglioma	4	L inferior/basal temporal	3	0	1	F7,C3,T3,T5	0.62	0.02			C3,F7,T3,T5	0.15	0.10
8	1, f	polymicrogyria, FCD 1a	450	R dorsal lateral prefrontal	20	450	5	C4,F4,F8,Fp2	11.18	2.72	4.17	0.00			
9	3, m	FCD 2a	30	R dorsal lateral prefrontal	44	0	1				1.80	0.00	C4,F4,F8,Fp2	0.53	0.37
10	6, f	angiocentric glioma	0.5	R dorsal lateral prefrontal	29	0.3	5				2.79	1.24	C4,F4,F8,Fp2	0.63	0.44
11	17, m	perinatal ischemic lesion	2	R lateral posterior temporal & lateral occipital	38	0	1						O2,P4,T4,T6	0.08	0.07

Seizure etiology, pre- and postsurgical seizure frequency, resection, follow-up duration, final seizure outcome, HFO recording channels and rates in the pre- and postsurgical scalp EEG or ECoG. m: male; f: female; L: left; R: right; FCD: focal cortical dysplasia; DNET: Dysembryoplastic neuroepithelial tumor; ECoG: electrocorticography; HFO: high frequency oscillations.

**Figure 3:**
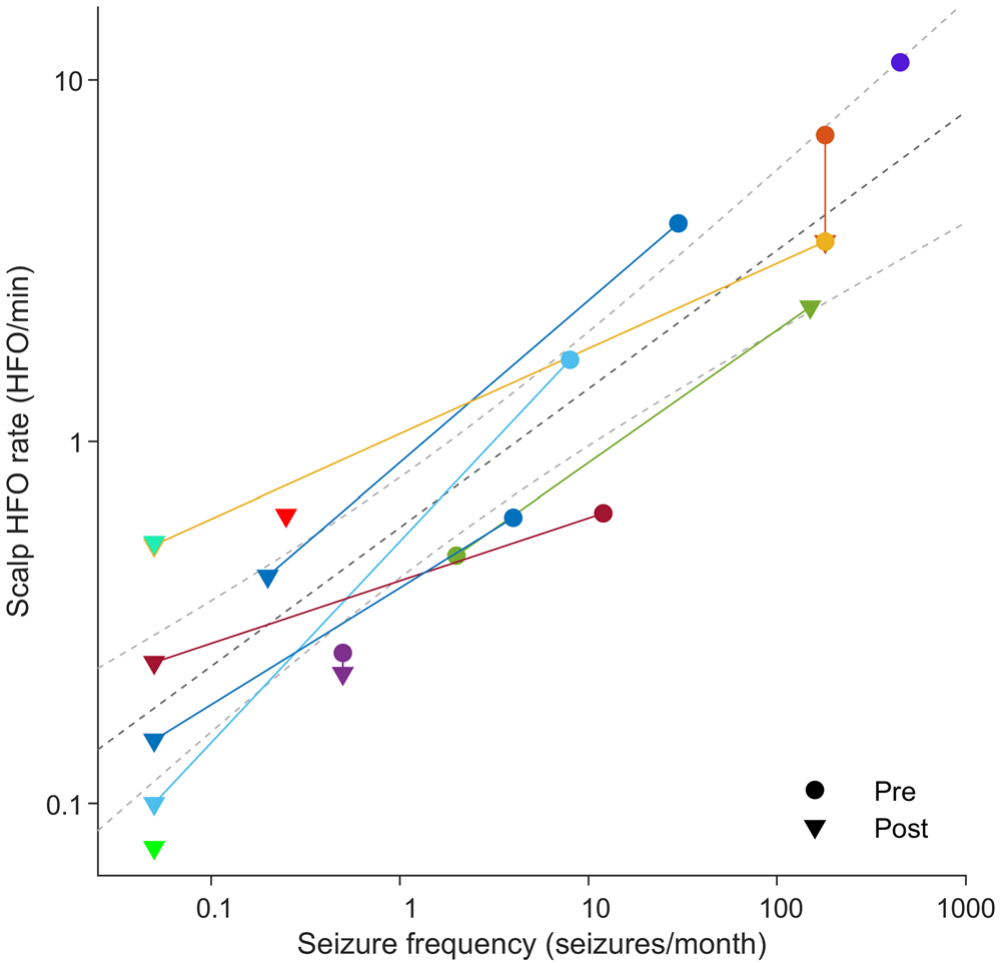
Scalp HFO rate mirrors seizure frequency. Scalp EEG recordings before (circles) and after (triangles) epilepsy surgery were pooled across our patient cohort (colors denote different patients). Axes are in logarithmic scale. For illustrative purposes, seizure freedom was approximated by 0.05 seizures/month. The HFO rate in the scalp EEG correlated with seizure frequency (R^2^ = 0.80, *p* < 0.001, linear regression).

Scalp HFO rates over the affected hemisphere exceeded the threshold of 0.25 HFO/min in 14 EEG recordings, all but two from patients with active epilepsy (PPV = 86%) and lay below this threshold in three recordings, two of which were from seizure-free patients (NPV = 80%, accuracy = 84% CI [62% 94%]). Overall, scalp HFO rates were higher in patients with active epilepsy (19 recordings, *p* = 0.0066, Wilcoxon’s ranksum test) and decreased following successful epilepsy surgery. Intra-individual decrease in HFO rate between pre- and postsurgical recordings mirrored decrease in seizure frequency (8 cases, χ^2^1 = 8, p = 0.0047). For all 19 recordings, scalp HFO rate over the affected hemisphere correlated with seizure frequency (R^2^ = 0.80, *p* < 0.001, linear regression), as illustrated in Fig. 3.

### Scalp HFO rates respond to surgical therapy

The decrease in scalp HFO rate on the affected hemisphere corresponded to a decrease in seizure frequency following the full resection of the epileptogenic zone in patients 5, 6 and 7 (1.68 to 0.10 HFO/min, 0.63 to 0.25 HFO/min and 0.62 to 0.15 HFO/min), and the partial resection of the epileptogenic zone in Patient 1 (4.01 to 0.43 HFO/min). The increase in scalp HFO rate (0.48 HFO/min to 2.36 HFO/min) reflected an increase in seizure frequency in Patient 4 that failed to respond to surgery due to an underlying genetic defect. Postsurgical scalp HFO rate was particularly low (0.10 HFO/min & 0.08 HFO/min) in two patients that achieved seizure freedom (Patient 5 & 11) and particularly high (3.57 HFO/min & 2.36 HFO/min) in two patients that remained unaffected by surgery (Patient 2, surgery 1, & Patient 4).

The focal skull defect after a craniotomy may increase the amplitude of fast rhythms, thus affecting the rate of HFO detected in the scalp EEG. However, we did not observe a typical breach rhythm in any of our scalp EEG recordings. Furthermore, the focal skull defect is expected to result in an increased HFO rate, whereas we observed a reduced HFO rate in 7 of 8 cases. Also, in the only patient with an increased HFO rate, no breach rhythm was recorded, whereas the postsurgical increase in seizure frequency offers a better explanation for the increased HFO rate in this case.

### The LNA improves HFO detection

To quantify the benefit of the LNA, we compared simultaneous CD and LNA recordings in Patient 2, as shown in Fig. 2. The spectral power followed a 1/f trend (Fig. 2a), typical for scalp EEG. Above 100 Hz, the LNA achieved a lower noise level, as expected given the lower internal noise level of the amplifier. Due to the improved SNR (Fig. 2c,f), the amplitude threshold of the detector in the ripple band Th_amp_ was lower for the LNA (1.07 ± 0.27 µV) than for the CD (1.30 ± 0.12 µV) across all channels and 5 min epochs (*p* < 0.001, Wilcoxon matched-pairs signed rank test). This enabled the detection of lower-amplitude HFO (mean amplitude: LNA 3.89 ± 2.41 µV vs. CD 5.26 ± 2.34 µV, *p* < 0.001, Wilcoxon rank sum test), thus increasing the yield of HFO detection (HFO rate in the affected hemisphere: LNA 8.20 ± 3.58 HFO/min vs. CD 5.15 ± 2.51 HFO/min, *p* < 0.001, Wilcoxon matched-pairs signed rank test, Fig. 2d). To compare artifacts in LNA and CD, we carefully examined all putative HFO in this patient. During visual inspection, similar numbers of putative HFO were rejected as artifacts for LNA (42/823 = 5%) and CD (36/535 = 7%). Of the 781 LNA HFO, 341 (44%) were also detected by CD. The LNA thus widened the population of HFO that could be detected and analyzed.

Next, we compared the clinical utility of LNA and CD recordings over the group of patients. There were 15 simultaneous recordings available. We first performed automated HFO detection in LNA and CD recordings separately. Then we visually rejected artifacts in LNA recordings. For events detected by both LNA and CD, we used the results of visual artifact rejection for LNA. Finally, we visually rejected artifacts in CD for events detected only by CD and not by LNA. HFO rates were lower for CD than for LNA (2.21 HFO/min vs 1.57 HFO/min, *p* < 0.001, Wilcoxon matched-pairs signed rank test). Over these 15 CD recordings, scalp HFO rate over the affected hemisphere correlated with seizure frequency (R^2^ = 0.78, *p* < 0.001, linear regression). Scalp CD HFO rates were higher in patients with active epilepsy (15 recordings, *p* = 0.0080, PPV = 80%, NPV = 60%, accuracy = 73% CI [48% 89%]). While the LNA widened the population of HFO that could be detected, both CD and LNA recordings yielded a similar prediction of seizure activity with our HFO detection algorithm.

### HFO in scalp EEG are associated with HFO in ECoG

The location of the highest HFO rates in scalp EEG corresponded to the location of the highest HFO rates in ECoG. HFO rates in the presurgical scalp EEG of Patient 8 with a right frontal epileptogenic zone, were higher over the frontal electrodes of the affected hemisphere compared to the non-affected hemisphere (11.18 vs 2.72 HFO/min) (Table 1), in line with the high HFO rates over the right frontal lobe in pre-resection ECoG. In Patient 1 with a lateral/mesial occipito-temporal epileptogenic zone, HFO rates in the presurgical scalp EEG were high in the occipital contacts of both hemispheres (4.01 and 3.48 HFO/min). Presurgical scalp HFO localization adjacent to the midline was in line with the particularly high HFO rates on the occipital lobe in pre-resection ECoG (31.73 HFO/min).

HFO rates in the postsurgical scalp EEG of Patient 1 with a right temporo-occipital epileptogenic zone, were significantly reduced compared to presurgical scalp EEG, but still higher over the affected hemisphere (0.43 vs 0.18 HFO/min), in agreement with the presence of residual HFO in post-resection ECoG (4.62 HFO/min). HFO rates in the postsurgical scalp EEG of Patient 10 with a right frontal epileptogenic zone, were higher over the affected hemisphere (0.63 vs. 0.44 HFO/min), in line with the presence of residual HFO in the post-resection ECoG (1.24 HFO/min).

## Discussion

To our knowledge, we are the first to show that scalp HFO rates mirror seizure frequency, and thus epilepsy severity, in pediatric drug-resistant focal epilepsy. Moreover, in consecutive scalp and invasive recordings, we show the response of HFO rates to successful surgical treatment in this patient group. In this study, we demonstrate the feasibility of recording HFO on scalp EEG and establish a direct correlation of scalp HFO to invasive HFO over time. Our findings suggest that non-invasively detected scalp HFO may represent a critical resource for clinical assessment in a broad population of patients affected by epilepsy.

### HFO rate in scalp EEG corresponds to disease severity

In our study, scalp HFO rate in pediatric drug-resistant focal epilepsy correlated with seizure frequency, and thus with epilepsy severity, in the individual patient. This observation is in line with a previous study that addressed the benign electroclinical syndrome of idiopathic focal (Rolandic) epilepsy, showing that HFO rates in scalp EEG, but not spike rates, correlate with seizure frequency^12^. Our study has not only corroborated this observation but also extended its applicability to the highly refractory population of children and adolescents with lesional focal epilepsy, in response to the urgent need for readily accessible biomarkers of drug or therapy efficacy in this patient subgroup. Furthermore, in contrast to previous studies that considered mainly infants and young children^10–12,25^, thus profiting from the decreased signal attenuation due to the thinner skull bone^26,27^, our study population ranged from infancy to late adolescence, demonstrating the feasibility and applicability of this measure to a broader age group.

### Scalp HFO rate responds to surgical therapy

HFO rate in scalp EEG decreased following successful epilepsy surgery, thus highlighting HFO as an outcome biomarker in this setting. The response of scalp HFO rate, a marker of disease severity, to treatment has been previously shown in infants with West syndrome undergoing adrenocorticotropic hormone (ACTH) treatment^10^ as well as in young children with atypical benign partial epilepsy undergoing methylprednisolone treatment^11^. We are extending this observation to children and adolescents with drug-resistant focal epilepsy undergoing surgical treatment, following the evolution of HFO rates over consecutive recordings before, during, and after epilepsy surgery. In our study, we have shown that the resection of the cortical HFO generators results in a reduction of HFO rates recorded in scalp EEG, irrespective of the localization or depth of these HFO generators. In contrast to a recent study supporting that HFO are only visible in scalp EEG with superficial lesions^28^, we recorded scalp HFO associated with deep-seated lesions. However, scalp HFO rates corresponding to deep-seated lesions were much lower than those associated with superficial generators. Infants and young children showed overall higher HFO rates compared to older children and adolescents, most probably due to the higher skull conductivity in the first years of life, in line with a previous study comparing children and adults^10^. This feature underlines the potential of scalp HFO as a marker of disease severity and treatment response, particularly in the pediatric population.

### The LNA improves scalp HFO detectability

The implementation of the low-noise amplifier in our study resulted in an improved SNR for the single event and thus a significant increase in the number of HFO detected compared to a commercially available medical device. In the high-frequency domain, the EEG noise is dominated by technical factors, proportional to the electronic noise of the amplifier and to the impedance of recording electrodes. Our amplifier features an electronic noise ten times lower than that of a CD. When combined with impedance as low as 1 kΩ, it substantially reduces the background noise level. The benefit of LNA technology has been previously demonstrated in the ECoG for ripples and FR^19,20^, and in the scalp EEG for evoked responses up to 1 kHz^29,30^. In our study, we have provided evidence for the feasibility and validity of this novel methodology in scalp HFO detection, thus extending its applicability. Due to the use of the LNA, the scalp HFO rates recorded in our study (mean HFO rate over the affected hemisphere: 2.65 HFO/min) were considerably higher than the previously reported scalp ripple rates in studies performed with conventional amplifiers (0.4–0.8 HFO/min)^7,8,31^. Nevertheless, in the direct comparison over simultaneous CD and LNA recordings over the group of patients, both amplifiers yielded a similar prediction of seizure activity within our automated HFO detection algorithm.

### The automated detection of scalp HFO is feasible

For this study, we used the prospective definition of HFO implemented in our previously developed invasive HFO detector that has been validated against seizure outcome and adapted it to the features of the scalp EEG. The application of the automated detector resulted in a consistent correlation between the location of scalp HFO and the underlying epileptogenic lesion and, furthermore, between the location of scalp HFO and invasive HFO. Moreover, the rate of the detected scalp HFO correlated with the state of the disease. While the relevance of HFO is increasingly recognized, their use as a biomarker in clinical practice is limited by the absence of reliable automated tools to replace the current gold standard of visual HFO analysis that is not only very time-consuming but can also be subjective. So far, detection algorithms have only rarely been validated against postsurgical seizure freedom^2–4,22^. However, to develop sophisticated algorithms for HFO detection and thus establish the clinical utility of HFO as a biomarker for epileptic tissue, we need to prospectively define HFO whose removal predicts seizure freedom and train the algorithms towards their identification. This crucial process is complex yet feasible, as demonstrated in recent studies from our group^3,4^. The automated identification of clinically relevant HFO based on their prospective, bias-free definition, is the prerequisite for their broad utilization as a valid EEG biomarker in invasive as well as in scalp EEG recordings.

Interictal scalp FR have shown a correlation to disease activity in only one study so far^26^. This study reported particularly low scalp FR rates and authors suggested caution in the interpretation of their findings, since non-cephalic signals in the scalp EEG (e.g. paroxysmal stereotyped artifacts or muscle activity) pose a risk for the detection of “false FR”^32^. Our recordings with evoked HFO point to a low signal-to-noise ratio for scalp FR^33^. Taken together with our unfavorable findings regarding scalp FR, we do not consider scalp FR to have clinical relevance in the data recorded with our setup.

### HFO in scalp EEG are associated with HFO in ECoG

In our study, pre- and postsurgical scalp ripples matched the location and rates of pre- and post-resection FR in the acute intraoperative ECoG in the individual patient, thus establishing a link between scalp HFO and invasive HFO. This observation raises questions regarding the interrelations of HFO in the range of ripples and FR between the different EEG recording scales. So far, only two studies have reported on the relation between HFO detected invasively vs. non-invasively. Zelmann *et al*.^16^ has demonstrated a low correspondence of ripples between scalp EEG and subdural ECoG, whereas Kuhnke *et al*.^9^ showed that scalp EEG ripples mostly point to the same lobe as the ripples in subdural EEG. In the intraoperative ECoG, FR are highly specific in predicting seizure outcome^2,3,20,22^. In the long-term presurgical invasive EEG, it has been shown that the resection of simultaneously occurring ripples and FR is the most accurate predictor of seizure outcome in the individual patient^4^. However, it should be noted that the distinction between ripples and FR has mainly historical reasons. Rather, the signature of clinically relevant HFO seems to be the oscillatory energy that extends from the ripple band into higher frequencies^4,34^. Of these clinically relevant HFO, the spectral component in the ripple band has a higher SNR and is thus more likely to be detected in scalp EEG. We, therefore, propose that the clinically relevant HFO recorded in ECoG are a different aspect of the same pathophysiological entity that we have detected here as scalp HFO.

Since the generators of HFO, be it ripples or FR, are not yet agreed on, it cannot be ruled out that ripples and FR represent different phenomena and correspond to distinct features of epileptogenicity. Still, there is some evidence that the pathophysiological mechanism that generates HFO may yield ripples and/or FR as different aspects of the same phenomenon, depending on the recording setup. In subdural, intracranial ECoG as well as in stereotactic, intracerebral EEG, the instantaneous frequency spectrum of clinically relevant HFO events is usually continuous without prominent troughs^4^. The distinction between R and FR may thus be a consequence of the frequency band filtering. In the results presented here, pre- and postsurgical scalp ripples matched the location and rates of pre- and post-resection FR in the acute intraoperative ECoG in the individual patient, thus establishing a link between scalp HFO and intracranial HFO. We have thereby provided evidence for a relation between scalp EEG ripples and ECoG FR in our pediatric cohort.

### Future directions

While LNA technology permits to improve the SNR for specific scalp electrode positions, a significant requirement is optimal spatial sampling^18,31,35–38^. High-density scalp EEG (HD-EEG) is gaining ground in the last decade, as it can be readily implemented in every epilepsy unit, drastically increasing the resolution of scalp recordings and thus facilitating the matching of scalp signals to their cortical correlates^39,40^. Indeed, HD-EEG has recently been shown to increase the yield of HFO detection compared to a standard 10–20 montage and provide a more accurate localization of the seizure onset zone in children and adults undergoing epilepsy surgery^31^.

Magnetoencephalography (MEG) is another non-invasive modality that provides high-density coverage of the entire brain while remaining unaffected by issues of skull conductivity and artifact contamination^36^. Indeed, ripples have been recently detected in MEG after beamforming^37^ and validated against invasive EEG^38^ and simultaneous scalp EEG^35^. The added value of MEG compared to HD-EEG regarding specific locations of cortical HFO generators, i.e., tangential to the cortical surface, remains to be investigated. Combined approaches in LNA EEG/MEG systems have been shown to enable the bimodal non-invasive detection of spike-like human somatosensory evoked responses at 1 kHz^41^. The integration of LNA in HD-EEG and MEG in the clinical setting may prove the optimal setup for the non-invasive detection of HFO.

HFO analysis in routine, non-invasive, scalp EEG has opened a new study field, broadening the range of patients with epilepsy that can be assessed beyond the subgroup with drug-resistant focal seizures undergoing an invasive evaluation or epilepsy surgery^1^. Non-invasive methods to evaluate disease activity and assess treatment response, such as scalp EEG, are in the spotlight of current research and are particularly promising in children since these present higher scalp HFO rates^10^. Considering the importance of timely intervention, particularly in early-onset epilepsy^42,43^, non-invasively detected HFO could serve to highlight a high risk for seizure onset and thus provide the indication for treatment initiation in vulnerable populations following early brain insults, e.g., hypoxic-ischemic encephalopathy or pediatric stroke.

## Conclusion

Scalp HFO rates mirror seizure frequency, and thus epilepsy severity, in pediatric drug-resistant focal epilepsy. The LNA considerably improves detectability, and the automated detector ensures a prospective, bias-free definition of clinically relevant HFO in scalp EEG. These findings are the first step towards using non-invasively detected scalp HFO for therapy monitoring in a broad population of patients affected by epilepsy.

## Supplementary information

Automated HFO detection

## Data Availability

The EEG data and HFO markings are freely available at https://gin.gnode.org/USZ_NCH/Scalp_EEG_HFO.

## Code availability

The code is freely available at the GitHub repository (https://github.com/ZurichNCH/Automatic-High-Frequency-Oscillation-Detector).
