# Publisher Correction: High-frequency oscillations in scalp EEG mirror seizure frequency in pediatric focal epilepsy

**DOI:** 10.1038/s41598-020-58621-3

**Published:** 2020-01-28

**Authors:** Ece Boran, Johannes Sarnthein, Niklaus Krayenbühl, Georgia Ramantani, Tommaso Fedele

**Affiliations:** 1 Klinik für Neurochirurgie, UniversitätsSpital & Universität Zürich, Zürich, Switzerland; 2 Zentrum für Neurowissenschaften Zürich, ETH Zürich, Zürich, Switzerland; 3 Pädiatrische Neurochirurgie, Universitäts-Kinderspital Zürich, Zürich, Switzerland; 4 Neuropädiatrie, Universitäts-Kinderspital Zürich, Zürich, Switzerland; 5 Institute of Cognitive Neuroscience, Higher School of Economics - National Research University, Moscow, Russian Federation

Correction to: *Scientific Reports* 10.1038/s41598-019-52700-w, published online 12 November 2019

In this Article, there is a typographical error in the Data availability section, where:

“The EEG data and HFO markings are freely available at https://gin.gnode.org/USZ_NCH/Scalp_EEG_HFO.”

should read:

“The EEG data and HFO markings are freely available at https://gin.g-node.org/USZ_NCH/Scalp_EEG_HFO.”

