# Supplementary material for: High-frequency oscillations in scalp EEG mirror seizure frequency in pediatric focal epilepsy
Source: Sci Rep. 2019 Nov 12;9:16560. doi: 10.1038/s41598-019-52700-w (PMC6851354; doi:10.1038/s41598-019-52700-w)
Supplement: Supplementary file 1 — Automated HFO detection [file 41598_2019_52700_MOESM1_ESM.docx]

**High-Frequency oscillations in scalp EEG mirror seizure frequency in pediatric focal epilepsy**

Ece Boran^1^, Johannes Sarnthein^1,2^, Niklaus Krayenbühl^1,3^, Georgia Ramantani^4*^, Tommaso Fedele^5*+^

^1^Klinik für Neurochirurgie, UniversitätsSpital & Universität Zürich, Switzerland

^2^Zentrum für Neurowissenschaften Zürich, ETH Zürich, Switzerland

^3^Pädiatrische Neurochirurgie, Universitäts-Kinderspital Zürich, Switzerland

^4^Neuropädiatrie, Universitäts-Kinderspital Zürich, Switzerland

^5^Institute of Cognitive Neuroscience, Higher School of Economics - National Research University, Moscow, Russian Federation

^*^These authors have contributed equally to this work.

^+^Corresponding author tofedele@hse.ru

Corresponding author: Tommaso Fedele

Institute of Cognitive Neuroscience, Higher School of Economics - National Research University, 101000, Moscow, 18 Myasnitskaya Ulitsa, Russian Federation; +7(495)772-95-90 , email: [tofedele@hse.ru](mailto:tofedele@hse.ru)

# Supplementary Information

## Automated HFO detection

HFO detection was performed with a previously validated automated detector^1-3^ (Supplementary Figure S1). The detection algorithm uses the instantaneous power spectrum as computed by the Stockwell transform in a three-stage workflow. In Stage I, the amplitude threshold (Th_amp_) is set in the HFO spectral range ^3^. Time intervals with high Stockwell entropy (low oscillatory activity) characterize the background activity. A low Th_amp_ indicates a low noise level in the signal, i.e., good signal quality. Events exceeding Th_amp_ are marked as events of interest (EoI). We defined the SNR of an event as the ratio of the root mean square amplitude of the filtered signal during the event and that during a 1 sec-window around the event. In Stage II, we selected all EoI that exhibited a high-frequency peak isolated from low-frequency activity in the time-frequency space. The number of EoI was further reduced in Stage III: for scalp EEG, EoI with very high amplitude (>20 µV) or low SNR (<2), and EoI co-occurring on both hemispheres were eliminated by automated artifact rejection. For ECoG, an EoI exceeding > 1 cm in extent was rejected as an artifact, since HFO are typically confined in a small patch of cortical tissue.

## Supplementary Figure S1. Workflow of HFO detection and artifact rejection in scalp EEG.


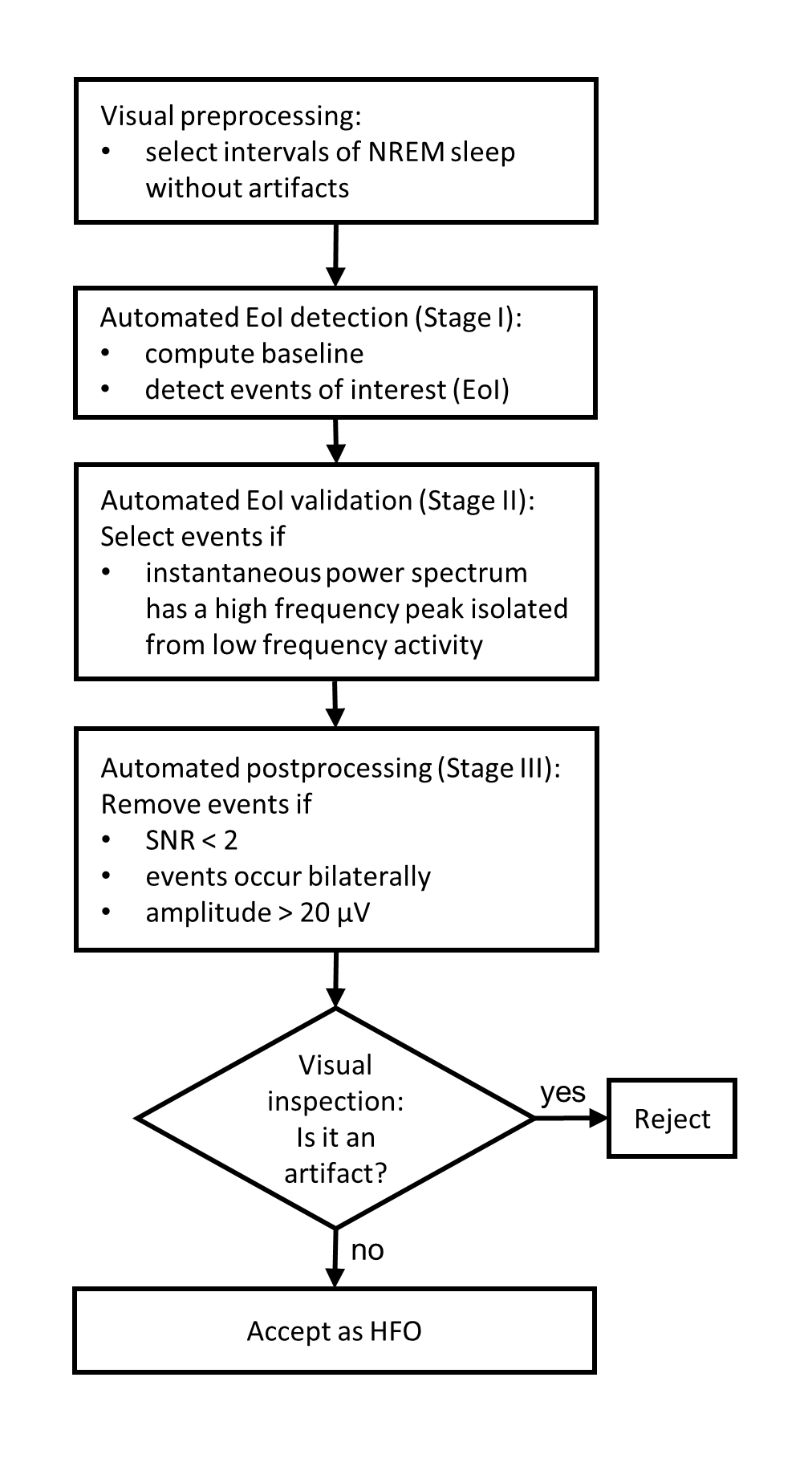


HFO analysis was performed with an automated detector, previously validated against seizure outcome for HFO in the intraoperative ECoG. In the workflow, human intervention was required only 1) for the selection of artifact-free signal intervals during NREM sleep and 2) for the exclusion of remaining artifacts after HFO detection. HFO detection was thus free from observer bias, allowing a prospective definition of a clinically relevant HFO.

# References

1 Fedele, T. *et al.* Prediction of seizure outcome improved by fast ripples detected in low-noise intraoperative corticogram. *Clin Neurophysiol* **128**, 1220-1226, doi:10.1016/j.clinph.2017.03.038 (2017).

2 Fedele, T. *et al.* Automatic detection of high frequency oscillations during epilepsy surgery predicts seizure outcome. *Clin Neurophysiol* **127**, 3066-3074, doi:10.1016/j.clinph.2016.06.009 (2016).

3 Fedele, T. *et al.* Resection of high frequency oscillations predicts seizure outcome in the individual patient. *Sci Rep* **7**, 13836, doi:10.1038/s41598-017-13064-1 (2017).
